# Predictive Value of Preoperative Profiling of Serum Metabolites for Emergence Agitation After General Anesthesia in Adult Patients

**DOI:** 10.3389/fmolb.2021.739227

**Published:** 2021-10-21

**Authors:** Qian Wang, Jiansuo Zhou, Taotao Liu, Ning Yang, Xinning Mi, Dengyang Han, Yongzheng Han, Lei Chen, Kaixi Liu, Hongcai Zheng, Jing Zhang, Xiaona Lin, Yitong Li, Jingshu Hong, Zhengqian Li, Xiangyang Guo

**Affiliations:** ^1^ Department of Anesthesiology, Peking University Third Hospital, Beijing, China; ^2^ Department of Laboratory Medicine, Peking University Third Hospital, Beijing, China

**Keywords:** emergence agitation (EA), postoperative delirium (POD), metabonomics, general anesthesia (GA), lipid metabolism

## Abstract

**Background:** Emergence agitation (EA) in adult patients under general anesthesia leads to increased postoperative complications and heavy medical burden. Unfortunately, its pathogenesis has not been clarified until now. The purpose of the present study was to explore the relationship between preoperative serum metabolites and EA.

**Methods:** We used an untargeted metabolic analysis method to investigate the different metabolomes in the serum of EA patients and non-EA patients undergoing elective surgical procedures after the induction of general anesthesia. A Richmond Agitation–Sedation Scale score ≥ +2 was diagnosed as EA during postoperative emergence. Non-EA patients were matched with EA patients according to general characteristics. Preoperative serum samples of the two groups were collected to investigate the association between serum metabolites and EA development.

**Results:** The serum samples of 16 EA patients with 34 matched non-EA patients were obtained for metabolic analysis. After screening and alignment with databases, 31 altered metabolites were detected between the two groups. These metabolites were mainly involved in the metabolism of lipids, purines, and amino acids. Analyses of receiver-operating characteristic curves showed that the preoperative alterations of choline, cytidine, glycerophosphocholine, L-phenylalanine, oleamide, and inosine may be associated with adult EA.

**Conclusion:** Multiple metabolic abnormalities (including those for lipids, purines, and amino acids) and other pathological processes (e.g., neurotransmitter imbalance and oxidative stress) may contribute to EA. Several altered metabolites in serum before surgery may have predictive value for EA diagnosis. This study might afford new metabolic clues for the understanding of EA pathogenesis.

## Introduction

“Emergence agitation” (EA) is defined as agitation surrounding awakening from general anesthesia. In the clinical setting, EA represents a fluctuating state of mental excitement and involuntary physical activity and may precipitate several clinical problems including dislodgement of indwelling catheters, self/staff injury, falling off in the operating theater/transport stretcher, etc. (Lee and Sung, 2020; Tolly et al., 2020). In particular, self-extubation may lead to serious consequences, that is, hypoxia and aspiration, if EA occurs and is not treated in a timely manner (Wei et al., 2020). Often, additional medical personnel are required to deal with EA patients, which increases the cost of postoperative management (Munk et al., 2016).

Pediatric EA has attracted widespread attention due to its high incidence (Voepel-Lewis et al., 2003; Silva et al., 2008; Kanaya, 2016; Menser and Smith, 2020). In recent years, adult EA has been the top priority of perioperative management (Lepousé et al., 2006; Lee and Sung, 2020). Accumulating evidence suggests that adult EA has a close relationship with a poor prognosis of postoperative recovery. Card and colleagues demonstrated that 60% of patients with EA developed the symptoms of delirium during different periods of postoperative recovery (Lepousé et al., 2006). A cohort study from Fields et al. indicated that EA was positively associated with the high incidence of postoperative delirium (POD) and pulmonary complications during the immediate postoperative period (Fields et al., 2018). Unfortunately, efficacious prophylaxis to reduce the risk of adult EA is still lacking. Therefore, it is pivotal to find both sensitive and specific biomarkers to facilitate the early recognition of EA.

A case-control study of 1950 patients from Kang and colleagues found that risk factors {cigarette smoking, male sex, age ≥65 years, body mass index (BMI) ≥24 kg/m2, poor physical condition [American Society of Anesthesiology (ASA) grade III/IV], intraoperative hemodynamic instability, and insufficient analgesia} were related to EA, whereas wound local anesthetic infiltration and dexmedetomidine application appeared to be protective factors against EA if undergoing thoracoscopic lung surgery (Kang et al., 2020). Another study indicated that a higher serum level of the brain-derived neurotrophic factor was associated with adult EA after gastrointestinal surgery (Mei and Tong, 2016). Recently, Wei et al. (2020) reviewed 18 studies and concluded that being male, smoking tobacco, having a urinary catheter, and postoperative pain were risk factors for EA, whereas age, use of inhalational anesthesia, history of substance misuse, and intraoperative use of benzodiazepines were possible risk factors for adult EA.

Metabolomics analysis (MA) is a rapidly developing approach to detect metabolite dynamics/alterations under external stimulation or interference. MA has huge advantages with regard to the prevention, diagnosis, treatment, and prognosis of the disease (Patti et al., 2012; Wang et al., 2016). Several studies have demonstrated that alterations in cerebrospinal fluid (CSF) or the serum level of metabolites are strongly associated with POD occurrence (Guo et al., 2017; Guo et al., 2019; Schaefer et al., 2019; Ida et al., 2020). Such studies have helped to elucidate the POD mechanism. Similarly, MA may also be used to advance our understanding of EA after the induction of general anesthesia. However, few studies have focused on preoperative metabolic profiling in adult EA patients.

We designed a case-control study to explore the correlation between the preoperative serum level of metabolites and postoperative EA. In the current study, we collected the serum samples of EA and non-EA patients before anesthesia and analyzed metabolic profiles based on liquid chromatography-tandem mass spectrometry (LC-MS/MS) to distinguish the metabolic differentiation between the two groups. We aimed to identify potential serological markers of early EA and to further explore EA pathogenesis.

## Methods and Materials

### Study Population

**FIGURE 1:**
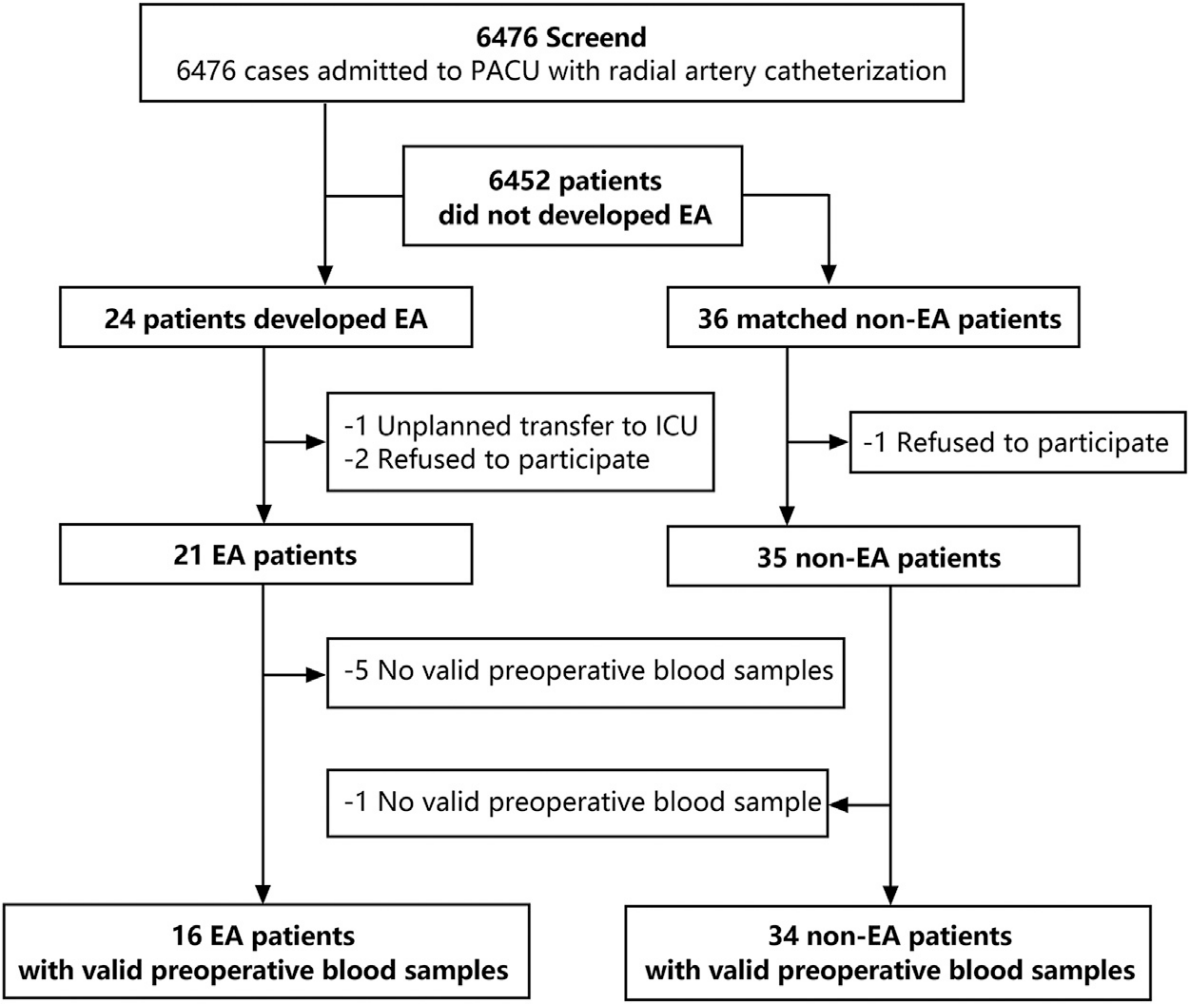
Flow chart of the research design. 6,476 patients were screened for emergency agitation, and 24 patients developed EA. 36 non-EA patients were matched with a ratio of 1:1.5 according to gender, age, BSA, and surgery types. Finally, 16 valid preoperative blood samples in the EA group and 34 valid preoperative blood samples in the non-EA group were collected for further analyses.

Patients (>18 years) undergoing elective surgery with general anesthesia who had an ASA physical class of I–III in Peking University Third Hospital between 1 June and 30 December 2020 formed the study cohort. Before the induction of anesthesia, all patients underwent catheterization of the radial artery. After surgery, patients recovered from general anesthesia in the postoperative care unit (PACU). A total of 6,476 patients were screened using the Richmond Agitation–Sedation Scale (RASS), and finally, 24 patients developed EA (Figure 1). To date, few studies provided accuracy with regard to EA morbidity because it varied due to different surgery types (1). In this study, 6,476 patients were screened and finally 24 patients incurred EA. Specifically, the match ratio varies from 1:1 to 1:4 in different metabolic studies (DeBose-Boyd, 2018; Han et al., 2020; Kimhofer et al., 2020). According to previous studies (Lee et al., 2017; Dizitzer et al., 2020), a match ratio of 1:1.5 was frequently used in case-control studies. Hence, we matched 36 non-EA patients based on sex, age, type of surgical procedure, and operation time of EA patients at a ratio of 1:1.5. In the EA group, one patient had an unplanned transfer to the intensive care unit (ICU) and two patients who refused to participate were excluded, and five invalid blood samples could not be used for analyses. In the non-EA group, one patient refused to participate, and one invalid blood sample was excluded. Finally, the preoperative blood samples of 16 EA patients and 34 non-EA patients were used for further analyses.

### Evaluation and Treatment of Emergence Agitation

Patients were transferred to the PACU for postoperative recovery. Then, the RASS (Table 1) was used while EA occurred. Calculation of the RAAS score was conducted by well-trained nursing staff. EA was defined as a RASS ≥ + 2 or dexmedetomidine utilization during the PACU stay (Tolly et al., 2020). If EA occurred, appropriate measures were taken immediately. Airway obstruction, hypoxia, inadequately treated pain, and hemodynamic instability were corrected immediately if they occurred (Munk et al., 2016). A low dose of propofol or midazolam was used for severe agitation, and repeat dosing was instigated until the patient could emerge calmly from general anesthesia (Demir and Yuzkat, 2018; Feng et al., 2019). All patients returned to the ward when they were fully awake and met the criteria for PACU discharge.

**TABLE 1:** Richmond Agitation and Sedation Scale (RAAS).

Score	Term	Description
+4	Combative	Overtly combative or violent; immediate danger to staff
+3	Very agitated	Pulls on or removes tube (s) or catheter (s) or has aggressive behavior
+2	Agitated	Frequent nonpurposeful movement or patient–ventilator dyssynchrony
+1	Restless	Anxious or apprehensive but movements not aggressive or vigorous
0	Alert and calm	
−1	Drowsy	Not fully alert but has sustained (>10 s) awakening
−2	Light sedation	Briefly (<10 s) awakens with eye contact to voice
−3	Moderate sedation	Any movement (but no eye contact) to voice
−4	Deep sedation	No response to voice but any movement to physical stimulation
−5	Unarousable	No response to voice or physical stimulation

### Sample Collection

Upon completion of pre-anesthesia preparation, 2 ml of arterial blood samples was collected after catheterization of the radial artery. Then, the samples were centrifuged at 3,000 rpm (1,000× g) for 10 min at 4°C to prepare serum, which was stored at −80°C until analysis.

### Collection of Clinical Data

The general information of eligible patients (age, sex, BMI, medication use, and allergy history) was collected. Laboratory tests (white blood cells, hemoglobin, platelets, aspartate aminotransferase, alanine aminotransferase, creatine, and albumin) and anesthesia/surgery information (type of surgical procedure, duration of surgery, volume of blood loss, blood transfusion, duration of anesthesia, and duration of PACU stay) were also collected from anesthetic records.

### Extraction of Metabolites

According to previous studies (Wang et al., 2016; Want, 2018), 100 μL of serum from each sample was added to 400 μL of extract solution (a mixture of methanol and acetonitrile, 1:1 v/v), including an isotopically labeled internal standard. Following sonication for 10 min in an ice–water bath, mixtures were vortex-mixed for 30 s and then incubated for 1 h at −40°C to precipitate proteins. After centrifugation at 12,000 rpm for 15 min at 4°C, an equal aliquot of all supernatants was mixed to prepare a quality-control sample for analyses.

### Profiling of Metabolites

LC-MS/MS was undertaken using an ultrahigh-pressure liquid chromatography (UHPLC) system (Vanquish™; Thermo Scientific, Waltham, MA, United States) with a UPLC BEH amide column (2.1 mm × 100 mm × 1.7 μm) coupled with a Q Exactive™ HFX Orbitrap mass spectrometer (Thermo Scientific) (Dunn et al., 2011). The mobile phase consisted of A (water phase: a mixture of 25 mmol/L ammonium acetate and 25 mmol/L ammonia hydroxide, pH = 9.75) and B (acetonitrile phase). The elution gradient was 0–0.5 min, 95% B; 0.5–7.0 min, 95%–65% B; 7.0–8.0 min, 65%–40% B; 8.0–9.0 min, 40% B; 9.0–9.1 min, 40%–95% B; 9.1–12.0 min, 95% B. The flow velocity of the mobile phase was set at 0.5 ml/min. The column temperature was 30°C. The auto-sampler temperature was 4°C, and the injection volume was 3 μL. The mass spectrometer was used to acquire MS/MS spectra on the information-dependent acquisition mode and to evaluate the full-scan MS spectrum continuously using Xcalibur™ (Thermo Scientific). The conditions for the electrospray ionization source were as follows: sheath-gas flow rate = 50 Arb; auxiliary-gas flow rate = 10 Arb; capillary temperature = 320°C; full MS resolution = 60,000; MS/MS resolution = 7,500; collision energy = 10/30/60; spray voltage = 3.5 kV (positive) or −3.2 kV (negative), respectively.

### Data Processing and Metabolite Identification

The raw data acquired from LC-MS/MS were converted to the mzXML format using ProteoWizard (http://proteowizard.sourceforge.net/). According to previous studies (Xiao et al., 2012; Ma and Chowdhury, 2013), ion annotation, spectral interpretation, and spectral matching were used to identify different characteristics of metabolites. In this study, an R (R Foundation for Statistical Computing, Vienna, Austria) program based on XCMS was used for detection, extraction, alignment, and integration of peaks. An in-house MS2 database named BiotreeDB (v2.1) was applied for metabolite annotation. The cutoff for annotation was set at 0.3 (Smith et al., 2006).

### Statistical Analyses

Data are the mean ± standard deviation (SD), proportions, and frequencies. The Kolmogorov–Smirnov method was used to test the normality of variables. Continuous variables following a normal distribution and skewed distribution are presented as the mean ± SD and interquartile range, respectively. Categorical variables are expressed as frequencies and proportions. Categorical variables with a normal distribution were tested using the two–independent-sample *t*-test. Categorical variables with a skewed distribution were evaluated using the Mann–Whitney *U*-test. A chi-square test was used to analyze categorical variables. SPSS 27.0 (IBM, Armonk, NY, United States) was employed for data analyses.

According to the protocol of metabolic phenotyping (Blaise et al., 2021), we used SIMCA-P (v15.0.2, Sartorius Stedim Data Analytics, Umea, Sweden) to analyze multivariate data of serum metabolites. A principal component analysis (PCA) model was conducted to visualize the different metabolomes between the EA group and the non-EA group. Also, an orthogonal partial least-squares discriminant analysis (OPLS-DA) model was used to evaluate a discrepancy between the two groups. A permutation test was used to verify the overfitting of this model, and two parameters were demonstrated to reveal the quality of this model. “R2” and “Q2” indicate the rate of model interpretation and the predictive ability of the model, respectively. Metabolites with variable importance in the projection (VIP >1) and *p* < 0.05 (Student’s *t*-test) were considered to be changed significantly. The Kyoto Encyclopedia of Genes and Genomes (KEGG) (www.genome.jp/kegg/) and MetaboAnalyst (www.metaboanalyst.ca/) were used for analyses of pathway enrichment. The receiver operating characteristic (ROC) curves were used to evaluate the predictive value of the selected metabolites.

## Results

### General Characteristics of Emergence Agitation Patients and Non–Emergence Agitation Patients

A total of 6,467 patients admitted to the PACU were screened for EA. Twenty-four patients developed EA (Figure 1). Another 36 non-EA patients matched for age, sex, and BMI were compared with EA patients. After removal of invalid samples, 16 patients in the EA group showed an RASS score of 3.56 ± 0.73, whereas 34 patients in the non-EA group had an RASS score of 0.03 ± 0.58 (*p* < 0.001). Statistical significance was not detected between the two groups with regard to age, sex, or BMI (Table 2).

**TABLE 2:** Patient characteristics and surgery types of the EA and non-EA groups.

Characteristics	EA group (*n* = 16)	Non-EA group (*n* = 34)	*p* value
RASS scores	3.56 ± 0.73	0.03 ± 0.58	*p* < 0.001
Age (year)	64.3 ± 13.9	64.5 ± 13.7	0.958
Age range	(31, 84)	(31, 85)	—
Male/female	13/3	28/6	1.000
BMI (kg/m^2^)	23.7 ± 3.4	23.7 ± 4.5	0.976
Surgery type			1.000
General	6	14	
Neurosurgical	2	4	
Thoracic	2	4	
Orthopedic	2	4	
Urologic	3	6	
Gynecologic	1	2	

RASS, Richmond Agitation–Sedation Scale; BMI, body mass index.

### Multivariable Analysis and Selection of Discriminant Variables

**FIGURE 2:**
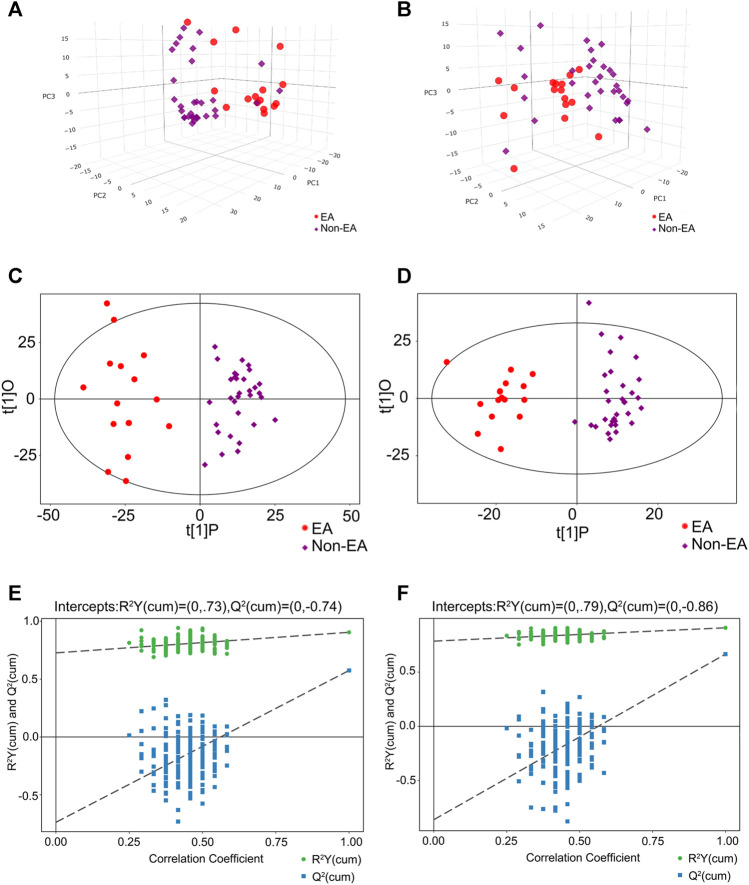
Multivariable analysis and selection of discriminant variables with both positive and negative ion modes between the EA group and the non-EA group. The PCA analyses were demonstrated as **(A)** (the positive ion model) and **(B)** (the negative ion model) between the two groups. **(C)** and **(D)** revealed the OPLS-DA score plots with further permutation tests [**(E)** in the positive mode and **(F)** in the negative mode] between the two groups.

An overview of metabolomic profiles was presented with PCA score plots of all samples in the positive (Figure 2A) and negative (Figure 2B) ion modes. This overview indicated an aggregate trend within groups and a discrete trend between the EA and non-EA groups. Similarly, OPLS-DA score plots were used to discriminate the multiple variable metabolites of all samples, from which remarkable separation of the two groups in the positive and negative ion modes can be seen in Figures 2C,D. Permutation tests were undertaken to evaluate the quantitation provided by this model (Figures 2E,F). Finally, R2Y (positive-ion mode, 0.73 and negative-ion mode, 0.79) and Q2 (positive-ion mode, −0.74 and negative-ion mode, −0.86) indicated a good fitting model.

### Differentially Expressed Metabolites Between the Emergence Agitation Group and the Non–Emergence Agitation Group

**FIGURE 3:**
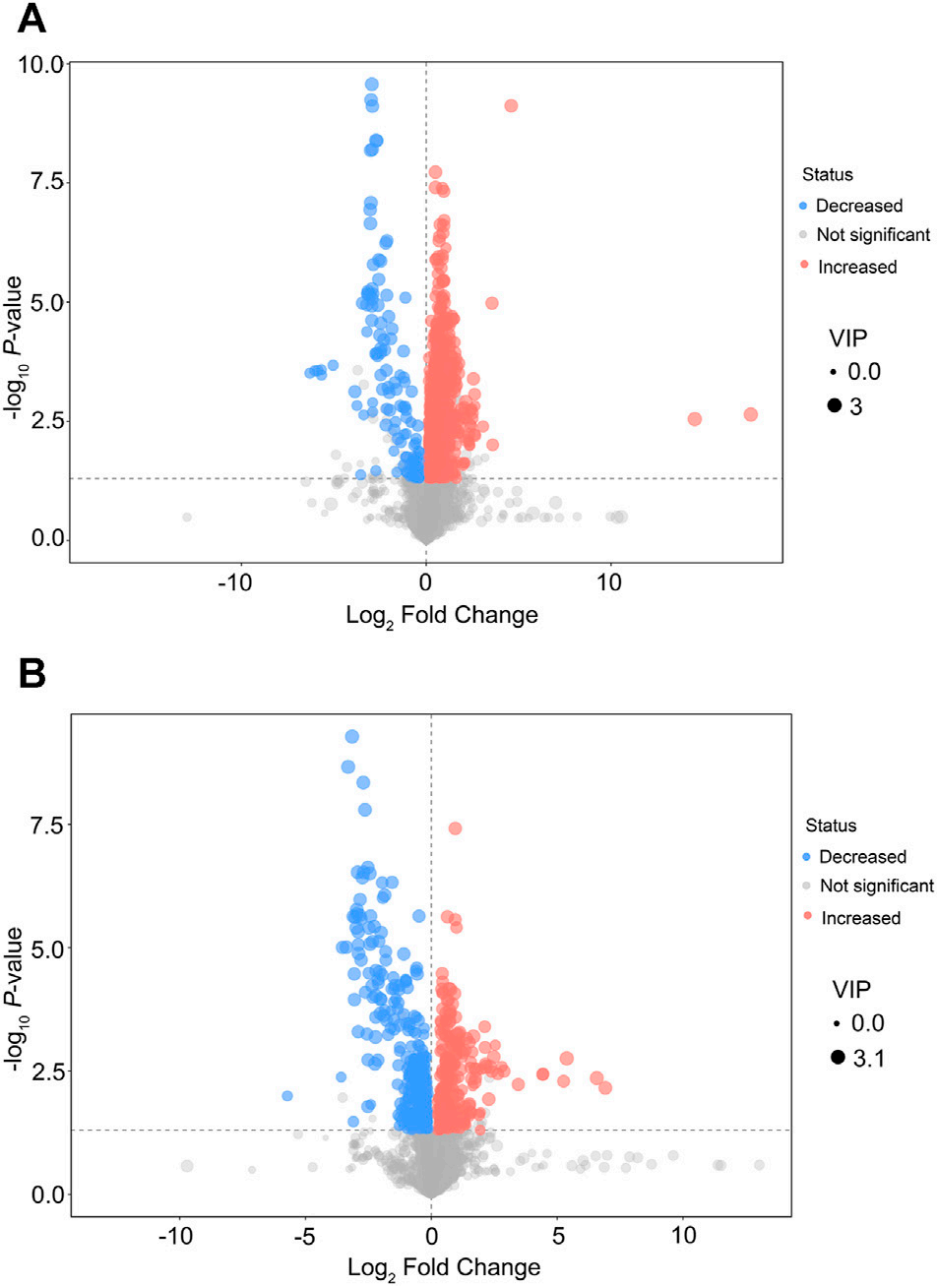
Volcano plots of differential features of metabolites between the EA group and the non-EA group. 1,108 features in the positive mode **(A)** and 586 features in the negative mode **(B)** were selected using the criteria of VIP > 1 and *p* < 0.05. As shown in the volcano plots, the increased and decreased features were marked as red and blue, respectively.

After multivariable analysis, the criteria of VIP >1 and *p* < 0.05 (Student’s *t*-test) were used to screen differentially expressed metabolites in serum. Finally, 1,108 features in the positive mode and 586 features in the negative mode were detected in serum samples in the EA group and the non-EA group, respectively. Volcano plots of different metabolites in the positive mode and the negative mode are presented in Figures 3A,B, respectively, in which increased features are marked blue and decreased features are marked red.

### Identification of Altered Metabolites and KEGG Pathways Analysis in Serum Between the Emergence Agitation Group and the Non–Emergence Agitation Group

A total of 164 metabolites were identified in the serum of cases in the EA group compared with that in the non-EA group. In sum, 135 metabolites (104 in the positive mode and 31 in the negative mode) were increased, and 29 metabolites (10 in the positive mode and 19 in the negative mode) were decreased. The KEGG annotation analysis found that these altered metabolites were involved in 33 KEGG pathways, of which metabolic pathways (*Homo sapiens*) are shown in Supplementary Figure S1.

**FIGURE 4:**
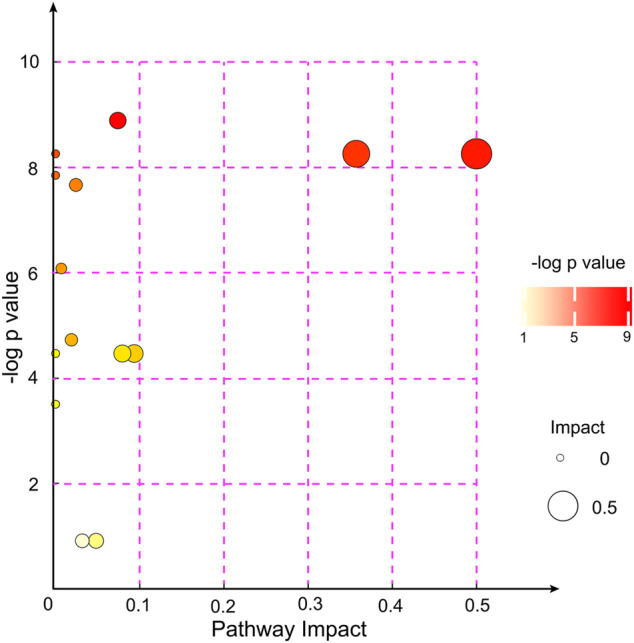
KEGG pathway enrichment analyses of the 31 altered metabolites between the EA group and the non-EA group. Each bubble represented a pathway, and 14 pathways were included in this bubble plot. The bubble size indicated the impact factor of the pathway in the topology analysis, and the bigger the size, the larger the impact factor. The bubble color represented the enrichment degree, and the deeper the color, the smaller the *p* value, indicating more significant enrichment.

After alignment of the metabolites with the MS database and screening, 31 significantly altered metabolites were selected to analyze the different metabolite patterns between the EA group and the non-EA group (Table 3). The differentially regulated metabolites were classified into several “superclasses”: “Lipids and lipid-like molecules” (11 metabolites), “Organoheterocyclic compounds” (Kanaya, 2016), “Organic acids and derivatives” (Munk et al., 2016), “Nucleosides, nucleotides, and analogs” (Wei et al., 2020), “Organic oxygen compounds” (Wei et al., 2020), “Organic nitrogen compounds” (Tolly et al., 2020), “Phenylpropanoids and polyketides” (Tolly et al., 2020), and “Alkaloids and derivatives” (Tolly et al., 2020). After alignment with the KEGG and MetaboAnalyst databases, altered metabolites were found to participate mainly in “purine metabolism,” “glycerophospholipid metabolism and nicotinate,” and “nicotinamide metabolism pathways.” We also analyzed the pathway enrichment of the 31 significantly altered metabolites, and 14 KEGG pathways were included (Figure 4), which were mainly associated with “Glycerophospholipid metabolism,” “Phenylalanine, yrosine, and tryptophan biosynthesis,” and “Phenylalanine metabolism” (Supplementary Table S1).

**TABLE 3:** Altered metabolites in the serum between the EA group and the non-EA group undergoing elective surgeries under general anesthesia.

Metabolites	ESI^+/−^	Super class	VIP	FC	*p*-value	*Q*-value	Trend
Allantoin	−	Organoheterocyclic compounds	2.87	4.47	0.003	0.018	Up
Theophylline	−	Organoheterocyclic compounds	2.18	4.39	0.001	0.010	Up
Cytidine	−	Nucleosides, nucleotides, and analogues	2.77	3.29	0.005	0.028	Up
Succinic acid semialdehyde	−	Lipids and lipid-like molecules	1.15	3.13	0.006	0.031	Up
D-2,3-Dihydroxypropanoic acid	−	Organic oxygen compounds	2.41	2.02	0.001	0.012	Up
Allopurinol	−	Organoheterocyclic compounds	1.97	0.41	<0.001	0.002	Down
Gamma-linolenic acid	−	Lipids and lipid-like molecules	1.93	0.20	<0.001	0.002	Down
Sedoheptulose	−	Organic oxygen compounds	2.50	0.20	<0.001	<0.001	Down
Inosine	−	Nucleosides, nucleotides, and analogs	2.69	0.13	<0.001	<0.001	Down
Tolmetin	+	Organic oxygen compounds	1.88	6.10	0.006	0.016	Up
Oleamide	+	Lipids and lipid-like molecules	2.16	5.33	0.004	0.013	Up
Glycerophosphocholine	+	Lipids and lipid-like molecules	2.62	4.12	0.002	0.009	Up
Trigonelline	+	Alkaloids and derivatives	1.44	4.05	0.024	0.038	Up
Decanoylcarnitine	+	Lipids and lipid-like molecules	1.45	2.67	0.026	0.040	Up
L-Cycl o(alanylglycyl)	+	Organoheterocyclic compounds	1.32	2.62	0.004	0.013	Up
L-alpha-Aspartyl-l-hydroxyproline	+	Organic acids and derivatives	1.35	2.58	0.021	0.036	Up
4,8 Dimethylnonanoyl carnitine	+	Lipids and lipid-like molecules	1.18	2.42	0.011	0.023	Up
Choline	+	Organic nitrogen compounds	2.68	2.31	<0.001	0.001	Up
1-Pyrrolidinecarboxaldehyde	+	Organoheterocyclic compounds	1.25	2.24	0.035	0.048	Up
2-Methylbutyroylcarnitine	+	Lipids and lipid-like molecules	2.09	2.17	<0.001	0.003	Up
PC(P-18:1 (11Z)/20:5 (5Z,8Z,11Z,14Z,17Z))	+	Lipids and lipid-like molecules	1.09	2.16	0.011	0.024	Up
Montecristin	+	Lipids and lipid-like molecules	2.45	2.07	<0.001	0.002	Up
l-Phenylalanine	+	Organic acids and derivatives	2.61	2.06	<0.001	<0.001	Up
Hypoxanthine	+	Organoheterocyclic compounds	1.76	0.47	0.002	0.008	Down
Isoleucyl-alanine	+	Organic acids and derivatives	1.35	0.37	<0.001	0.003	Down
Desglucocheirotoxin	+	Lipids and lipid-like molecules	2.29	0.23	<0.001	<0.001	Down
Isoleucyl-valine	+	Organic acids and derivatives	2.38	0.17	<0.001	<0.001	Down
Arabinosylhypoxanthine	+	Nucleosides, nucleotides, and analogs	2.17	0.13	<0.001	<0.001	Down
2-(3,4-Dihydroxybenzoyloxy)-4,6-dihydroxybenzoate	+	Phenylpropanoids and polyketides	2.34	0.11	<0.001	<0.001	Down
Sertindole	+	Organoheterocyclic compounds	1.27	0.02	<0.001	0.003	Down
8beta-Angeloyloxy-15-hydroxy-1alpha,10R-dimethoxy-3-oxo-11(13)-germacren-12,6alpha-olide	+	Lipids and lipid-like molecules	1.35	0.02	<0.001	0.002	Down

Note: ESI, electrospray ionization; VIP, variable importance in the projection; FC, fold change.

### Qualification for Potential Biomarkers to Predict Emergence Agitation in Serum Before Surgery

**FIGURE 5:**
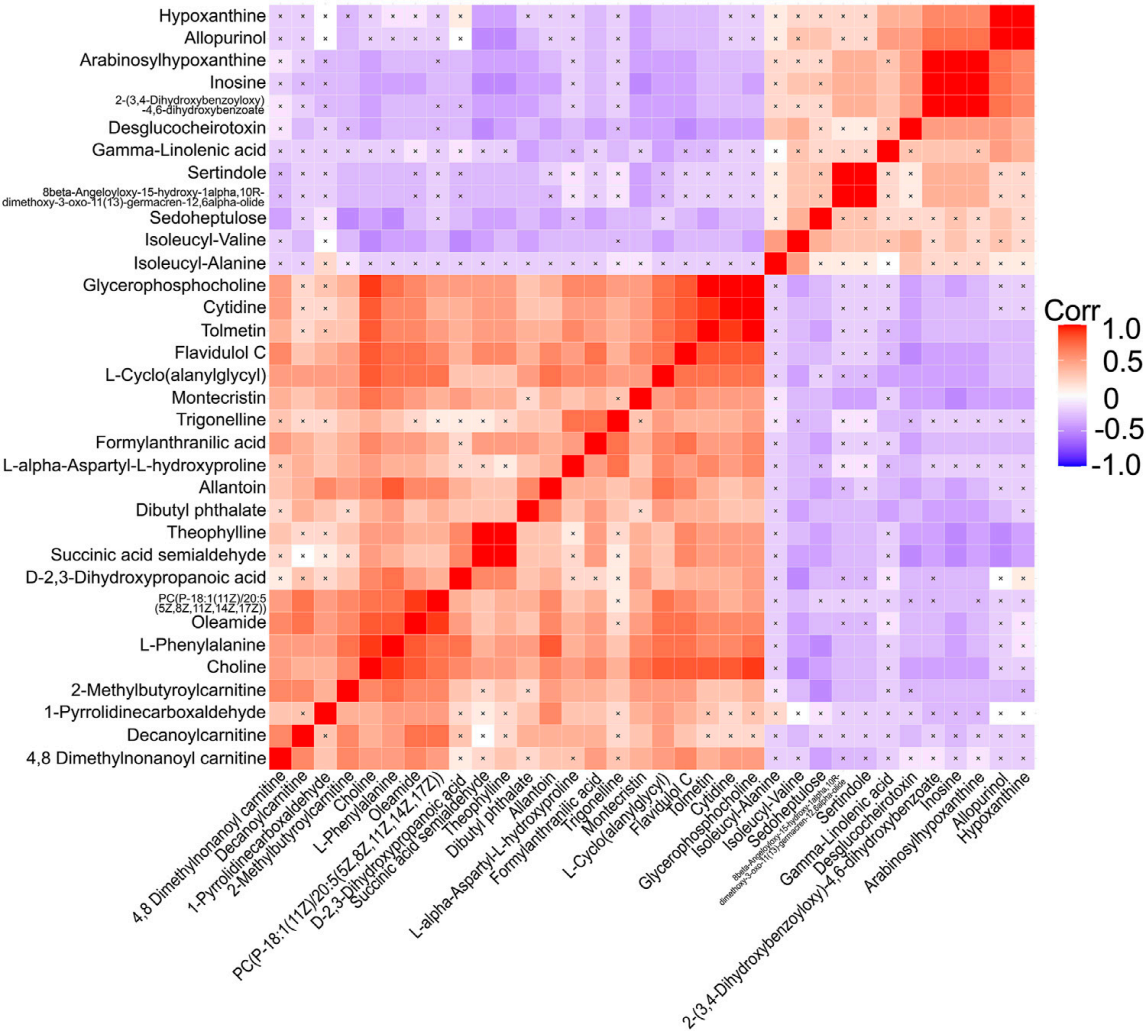
Correlation analyses of differential metabolites between the EA group and the non-EA group. 34 altered metabolites (including 3 metabolites of benzenoids) listed in Table 3 were performed Pearson correlation analyses using a heat map. The positive and negative correlations were shown in red and blue, respectively. A cross mark indicated a nonsignificant correlation.

**FIGURE 6:**
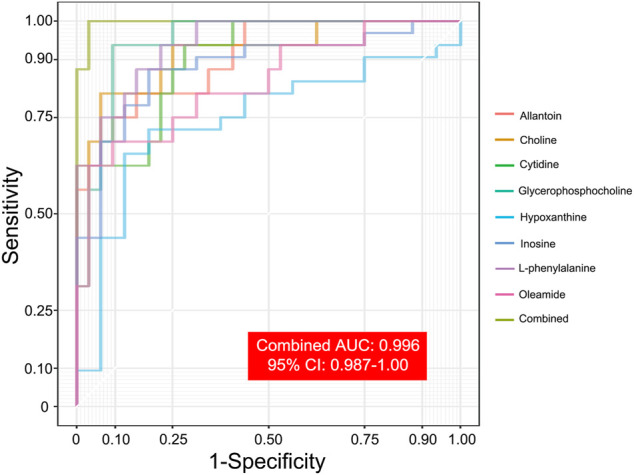
ROC analyses of selected metabolites for predicting EA occurrence. Eight selected metabolites with ROC analyses indicated the potential predictive value of EA occurrence. A combined AUC, marked as grass green, was 0.996 (95% CI: 0.987–1.00).

After correlation analyses (Figure 5), eight altered metabolites were found to be closely related to other metabolites. Choline, cytidine, glycerophosphocholine, L-phenylalanine, allantoin, and oleamide had a higher concentration while inosine and hypoxanthine had a lower concentration in the serum of EA patients than in that of non-EA patients. Next, we undertook analyses of the ROC curves of these metabolites (Figure 6), and a combined AUC (area under the ROC curve) was 0.996 (95% CI: 0.987–1.00). They all had an AUC over 0.7 (Supplementary Figure S2), which suggested that the chosen metabolites may be potential biomarkers for EA prediction.

## Discussion

The mechanism of EA pathogenesis is not known. A common hypothesis is that excessive reactions may occur due to internal or external stimuli as consciousness returns during the recovery from anesthesia (Brown et al., 2010; Tolly et al., 2020). It has been reported that several factors contribute to EA occurrence, including preoperative concomitant diseases and excessive sympathetic activation (Kim et al., 2019) from indwelling catheters and tubes (Kwon et al., 2016; Fields et al., 2018). Recently, Ma and colleagues suggested that high expression of inflammatory factors (e.g., tumor necrosis factor-α and interleukin-6) (Ma et al., 2017) after thyroid surgery indicated that neuroinflammation may participate in EA. Accumulating evidence has shown that EA/delirium has a strong relationship with POD (Sharma et al., 2005; Neufeld et al., 2013; Card et al., 2015). However, distinguishing EA from emergence delirium is still challenging (Kwak, 2010; Lee and Sung, 2020; Menser and Smith, 2020). Pediatric EA appears to be an asynchronism of different brain regions during the washout of volatile anesthetics and tends toward anxiety due to immature psychology (Dahmani et al., 2014).

LC-MS has obvious advantages with respect to repeatability, sensitivity, and coverage of metabolites and has become the most commonly used analytical method for high-throughput MA (Want, 2018; Lin et al., 2019). We used LC-MS/MS to differentiate the preoperative serum level of metabolites between EA and non-EA patients under general anesthesia. Finally, 34 differentially regulated metabolites (3 metabolites of benzenoids were not analyzed) were detected between the two groups, including lipid compounds, purine metabolites, and amino acids.

Choline plays a crucial role in maintaining the structural integrity of cell membranes (Zeisel et al., 2018). Choline can be converted to acetylcholine by acetyltransferase which, as a neurotransmitter, is involved in signal transmission in the neural system (Koelle, 1972; Tsetlin, 2020). Acetylcholine deficiency has been demonstrated in the brains of patients suffering from delirium (Tune and Egeli, 1999; Maldonado, 2013; Wang and Shen, 2018). In the present study, the serum level of choline in EA patients was increased (a fold-change of 2.31 compared with that in non-EA patients, *p* < 0.001). Jia and coworkers showed an increase in the choline level in the CSF of patients suffering from vascular dementia and a decrease in the acetylcholine level, which suggested that a decline in acetylcholine production may contribute to cognitive dysfunction (Jia et al., 2004).

Phosphatidylcholine is the main component of the lipid bilayer of cells. It also participates in fatty-acid metabolism, signal transduction, and substance transportation (Furse and de Kroon, 2015). Glycerophosphocholine is produced by phosphatidylcholine, which is derived from the cytidine 5′-diphosphocholine–choline pathway (Fernández-Murray and McMaster, 2005; Sonkar et al., 2019). In patients suffering from Alzheimer’s disease, a high level of glycerophosphocholine, phosphocholine, and choline in CSF has been detected (Walter et al., 2004). In acute and chronic neurodegenerative disease, an increased level of choline-containing phospholipids is probably due to cell membrane damage (Klein, 2000). Similarly, increases in lyso-phosphocholine (a soluble form released from phosphatidylcholine) and glycerophosphocholine concentrations in serum were also observed in EA patients in our study.

Oxidative stress has been found to be involved in the POD pathogenesis (Maldonado, 2013). Recently, Lopez et al. (2020) demonstrated that oxidative damage in the brain was ubiquitous after cardiac surgery in patients suffering from delirium, which can cause further neural injury and disruption to the blood–brain barrier (Lopez et al., 2020). Allantoin is a metabolic product of uric acid and is used as a marker of oxidative stress (Kozlik et al., 2020). In EA patients, we found a higher level of allantoin than in non-EA patients. A previous clinical study on delirium in the intensive care unit indicated that hyperuricemia was a predisposing risk factor of delirium occurrence (Sharma et al., 2012). Similarly, a case report demonstrated that a patient with gastric cancer who received chemotherapy developed delirium and hyperammonemia because the mutations of ORNT2 (ornithine transporter-2 for the urea cycle) and ETFA (electron transport flavoprotein alpha for fatty acid oxidation) exaggerated the response to the allopurinol challenge (Chu and Salzman, 2019), suggesting that uric acid metabolism dysfunction may contribute to delirium. In this study, we found an increase in allantoin in the preoperative serum of EA patients, indicating that uric acid metabolism may be involved in EA pathogenesis. Purine metabolism is part of the process of adenosine triphosphate degradation (which is an indicator of energy metabolism) (Garcia-Gil et al., 2018). We detected a decrease in the level of purine metabolites (e.g., hypoxanthine, inosine, and arabinosylhypoxanthine), which suggested energy metabolism dysfunction in EA patients before surgery.

Lipid metabolism is mainly responsible for the storage and supply of energy (DeBose-Boyd, 2018). Gamma-linolenic acid is an Ω-6 fatty acid and participates in the inflammatory response as a precursor of eicosanoids such as prostaglandins (Kapoor and Huang, 2006). We found that the level of gamma-linolenic acid was reduced in EA patients, indicating a weaker anti-inflammatory response during surgery. Oleamide is an endogenous fatty acid whose level is increased in CSF, which may induce “physiological sleep” (Boger et al., 1998; Mendelson and Basile, 2001). Oleamide has also been found to serve as an endogenous ligand for cannabinoid receptor-1 and to inhibit neurotransmitter (e.g., dopamine and gamma-aminobutyric acid) release (Fowler, 2004). An increase in the serum level of oleamide was observed in EA patients, which suggested that excessive inhibition of neurotransmitters may contribute to EA. Carnitine is essential for lipid catabolism, mainly through the promotion of coenzyme-A transport across the inner mitochondrial membrane for β-oxidation (Longo et al., 2016). Huo et al. suggested that a low concentration of three acylcarnitines in blood had a predictive value for Alzheimer’s disease and cognitive decline (Huo et al., 2020). An increase in the level of three carnitine derivatives (4,8-dimethylnonanoyl carnitine, decanoylcarnitine, and 2-methylbutyroylcarnitine) in the present study suggested that a high level of carnitines may be a risk factor of EA.

Phenylalanine is an essential amino acid. It can be converted to tyrosine through catalysis of phenylalanine hydroxylase, which involves the synthesis of monoamine neurotransmitters and catecholamines (Fernstrom and Fernstrom, 2007). Compared with non-EA patients, we discovered that the L-phenylalanine level was increased in the serum of EA patients, which suggested that disturbed synthesis of neurotransmitters may occur during EA development. Besides, levels of several peptides comprising amino acids, such as isoleucyl-alanine, isoleucyl-valine, and L-alpha-aspartyl-L-hydroxyproline, were also altered in the serum of EA patients. Whether these peptides are involved in EA pathogenesis is yet to be determined.

In this study, differential patterns of metabolites in the serum and possible metabolic abnormality between EA and non-EA patients were analyzed. But limitations still exist. First, the sample size was small due to the low prevalence of EA in adults. Second, we extracted metabolites only from serum; metabolites in CSF may be more helpful for exploring EA pathogenesis.

## Conclusion

We undertook a case-control study using a metabolic analysis method based on LC-MS/MS to uncover metabolite differences and to establish preoperative metabolic profiling of EA patients and matched non-EA patients. Preoperative abnormalities in the metabolism of lipids, purines, and amino acids may contribute to a vulnerable state in preoperative patients, thereby enabling EA development. Analyses of ROC curves for several metabolites were employed to further evaluate the sensitivity and specificity of predicting EA. Our study may provide a clue on the mechanism of EA pathogenesis in adult patients under general anesthesia. Verification of the molecular biology and metabolic function of altered metabolites should be undertaken in future research to deepen our understanding of how EA occurs.

## Data Availability

The raw data supporting the conclusions of this article will be made available by the authors, without undue reservation.
