# Supplementary material for: Predictive Value of Preoperative Profiling of Serum Metabolites for Emergence Agitation After General Anesthesia in Adult Patients
Source: Front Mol Biosci. 2021 Oct 21;8:739227. doi: 10.3389/fmolb.2021.739227 (PMC8566542; doi:10.3389/fmolb.2021.739227)
Supplement: Supplementary file 1 [file DataSheet1.docx]

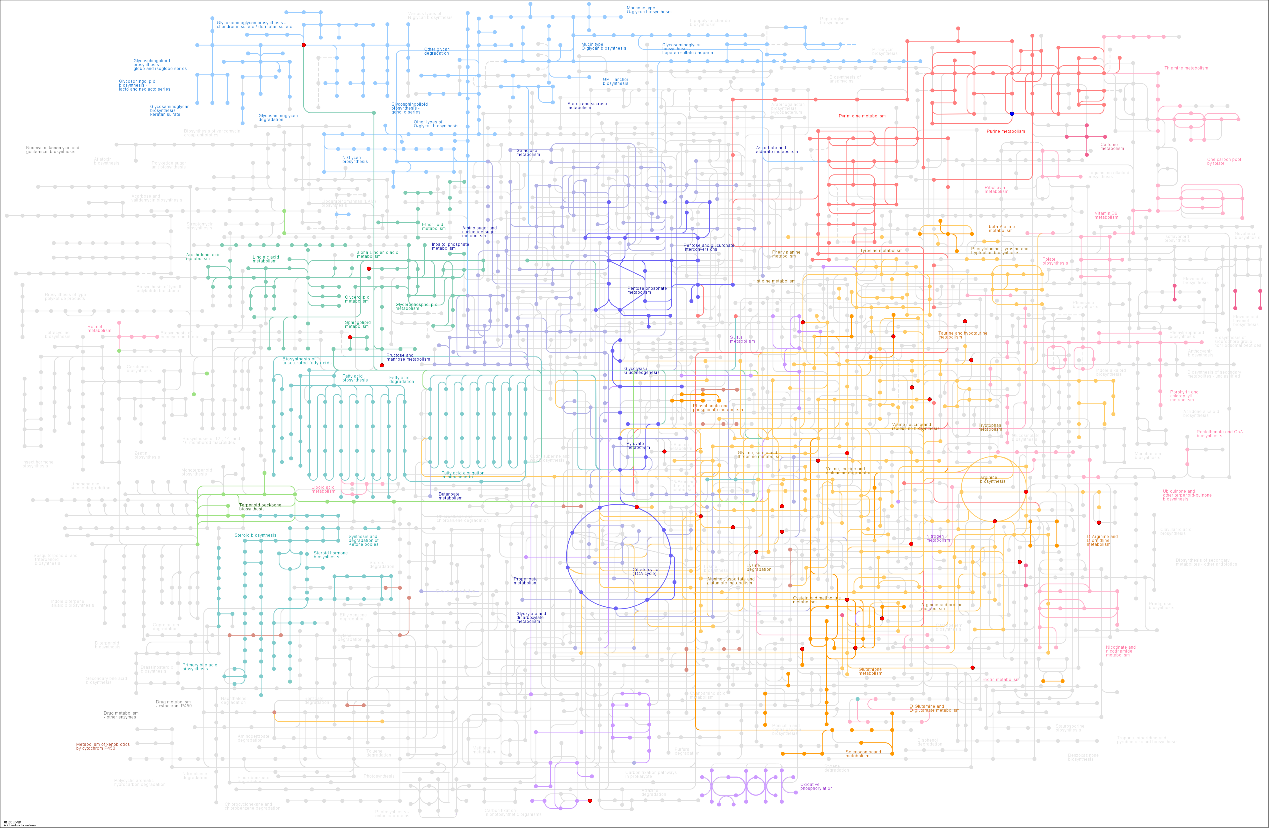


**Supplementary figure 1 KEGG metabolic pathways (Homo sapiens) map.** In this map, each dot represented a compound, red and blue dots representing the significantly up and down expressed compounds, respectively.


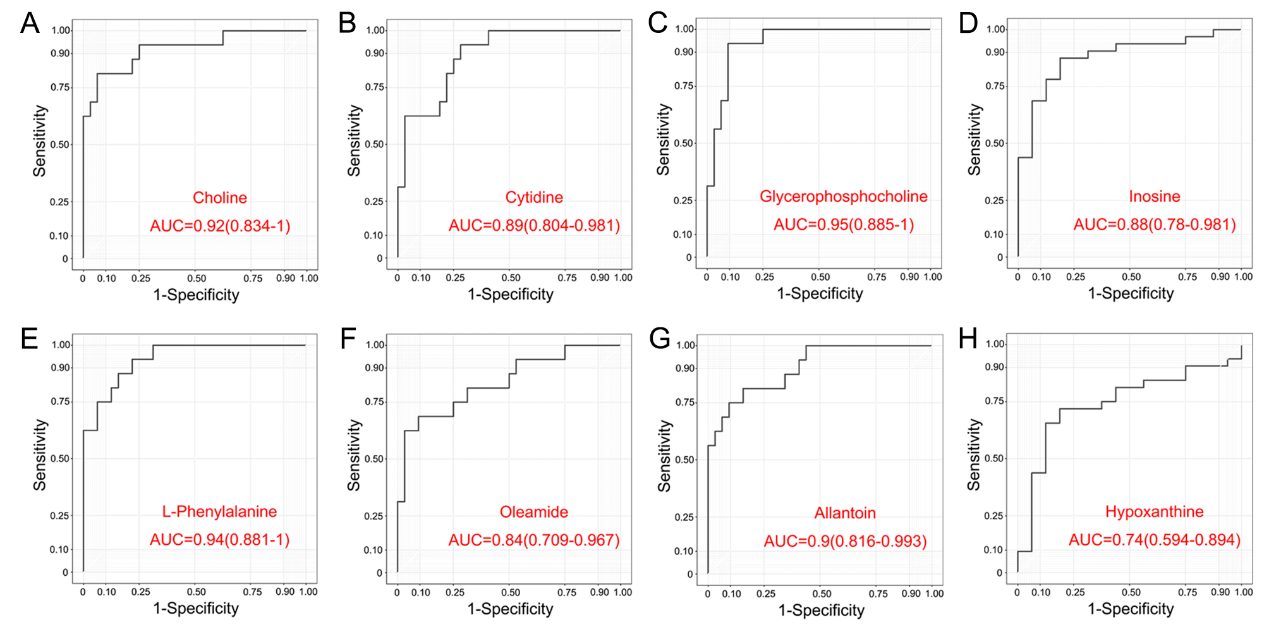


**Supplementary figure 2 ROC analyses of selected metabolites for predicting EA occurrence. A**, Choline, AUC = 0.92, CI (0.834-1); **B**, Cytidine, AUC = 0.89, CI (0.804-0.981); **C**, Glycerophosphocholine, AUC = 0.95, CI (0.885-1); **D**, Inosine, AUC=0.88, CI (0.78-0.981); **E**, L-Phenylalanine, AUC = 0.94, CI (0.881-1); **F**, Oleamide, AUC = 0.84, CI(0.709-0.967); G, Allantoin, AUC = 0.9, CI (0.816-0.993); H, Hypoxanthine, AUC = 0.74, CI (0.594-0.894).

Supplementary Table 1 KEGG pathway enrichment analysis of the 31 altered metabolites in the serum between EA group ang Non-EAgroup.

| Pathway Name | Match status | *P*-value | -log(p) | Impact |
| --- | --- | --- | --- | --- |
| Glycerophospholipid metabolism | 2/36 | 1.28E-09 | 8.8912 | 0.07396 |
| Phenylalanine, tyrosine and tryptophan biosynthesis | 1/4 | 5.50E-09 | 8.2598 | 0.5 |
| Phenylalanine metabolism | 1/10 | 5.50E-09 | 8.2598 | 0.35714 |
| Aminoacyl-tRNA biosynthesis | 1/48 | 5.50E-09 | 8.2598 | 0 |
| Ether lipid metabolism | 1/20 | 1.41E-08 | 7.8509 | 0 |
| Glycine, serine and threonine metabolism | 2/33 | 2.15E-08 | 7.6684 | 0.0242 |
| Pyrimidine metabolism | 1/39 | 8.28E-07 | 6.0819 | 0.0068 |
| Purine metabolism | 2/65 | 1.88E-05 | 4.7269 | 0.01885 |
| Glycerolipid metabolism | 1/16 | 3.41E-05 | 4.467 | 0.09346 |
| Glyoxylate and dicarboxylate metabolism | 1/32 | 3.41E-05 | 4.467 | 0.07937 |
| Pentose phosphate pathway | 1/22 | 3.41E-05 | 4.467 | 0 |
| Biosynthesis of unsaturated fatty acids | 1/36 | 0.000314 | 3.5027 | 0 |
| Alanine, aspartate and glutamate metabolism | 1/28 | 0.12311 | 0.90972 | 0.04808 |
| Butanoate metabolism | 1/15 | 0.12311 | 0.90972 | 0.03175 |
